# Announcements

**Published:** 1912-04

**Authors:** 


### BUFFALO MEDICAL JOURNAL

**A Monthly Review of Medicine and Surgery**

*EDITOR*

A. L. BENEDICT.

All communications, whether of a literary or business nature, books for review and exchanges, should be addressed to the editor, 354 FRANKLIN STREET, BUFFALO, N. Y Please make personal or telephonic calls before 1 p. m

THE BUFFALO MEDICAL JOURNAL will publish regularly. full transactions of any professional organization in western New York at cost. Personal notices, brief reports of society proceedings, and the like, are always welcome and will be published without charge.

Subscriptions may commence at any time. $2.00 per year in advance,

VOL. LXVII. APRIL, 1912 No. 9

*Sanitary Science is mainly applied decency.*

### Announcements

IT has become necessary to reduce the reserve file of back numbers still further. Anyone wishing old copies containing his own article, or any special report, obituary, etc., should apply immediately, stating exact date if possible, otherwise, approximate date. Libraries wishing complete files for binding should also apply promptly. We need a few copies of June 1911.

